# Critical care issues in adult liver transplantation

**DOI:** 10.4103/0972-5229.58535

**Published:** 2009

**Authors:** Palepu B. Gopal, Dharmesh Kapoor, Ravichandra Raya, M. Subrahmanyam, Deven Juneja, B. Sukanya

**Affiliations:** **From:** Departments of Anesthesia and Critical Care Medicine, and Gastroenterology, Global Hospital, Lak di-ka-pul, Hyderabad - 560 004, India

**Keywords:** Adult liver transplantation, critical care, orthotopic liver transplantation

## Abstract

Over the last decade, liver transplantation has become an operational reality in our part of the world. As a result, clinicians working in an intensive care unit are more likely to be exposed to these patients in the immediate postoperative period, and thus, it is important that they have a working knowledge of the common complications, when they are likely to occur, and how to deal with them. The main focus of this review is to address the variety of critical care issues in liver transplant recipients and to impress upon the need to provide favorable circumstances for the new liver to start functioning and maintain the function of other organs to aid in this process.

## Introduction

Liver transplantation is a treatment option for both acute liver failure (ALF) and end stage liver disease (ESLD). ALF is by far more morbid of the two states with acute and near-catastrophic multi-organ failure with the liver as the central and precipitating failed organ. Cirrhosis, on the other hand, is a slow and insidious liver dysfunction resulting in gradual failure of all other systems in the body. As a consequence of these scenarios, all the organ dysfunctions and failures are carried through the phase of transplant surgery and the posttransplant intensive care phase. The functioning transplanted liver forms the common denominator of organ system recovery and gives the patient a new life. Whereas, in the unfortunate event of graft dysfunction or failure of the transplanted liver, the patient will continue to be in multi-organ failure and will require continued or even an enhanced level of critical care support.

The unique pathophysiology of patients with ESLD has important implications for their critical care treatment, particularly in the postoperative state. Typically endotracheal intubation and mechanical ventilation are continued into the postoperative period. As a standard practice, all the modalities of monitoring and medication are continued into the postoperative period, the degree of which will be decided depending on the progress of the patient. In a very stable low risk transplant, all anesthetic and relaxant medications can be discontinued and patient can be fast tracked to weaning and extubation. Otherwise, the patient continues to be infused with longer acting anesthetics, analgesics, and muscle relaxants. In such cases, the process of weaning and time of extubation will depend on the patient's subsequent progress, which in turn is largely guided by ‘kick-starting’ of the engrafted liver. Intra-abdominal drains should be inspected for the nature and rate of blood and fluid loss. Biochemical, hematological and microbiological monitoring are implemented on a periodic manner depending on the protocol of individual units. Radiological investigations like chest X-ray, abdominal ultrasound, hepatic vascular Doppler monitoring are done on a minimum daily basis till the patient continues to require critical care services. Doppler ultrasonography has high sensitivity and specificity in evaluating hepatic artery, portal veins, hepatic veins, inferior vena cava, and the bile duct for signs of thrombosis or stenosis in a posttransplant patient.

Though it is the degree of functioning of the transplanted liver that largely determines the other system functions, their dysfunction may be an outcome of an independent entity, not least important among which is sepsis. Optimum renal function is of paramount importance as a determinant of good outcome.[1]

## Function of the Liver Allograft

A smooth ICU course after liver transplant is dependent on satisfactory graft function, which can be assessed by clinical parameters, such as wakefulness, normal mentation, improvement of muscle-power, stable respiratory effort, change in drain fluid from sero-sanguinous to ascites, improvement in urine-output, and lab parameters including improvement in acidemia, stable platelet counts, stable and improving INR without use of fresh-frozen plasma, improving serum lactate, declining transaminases, and normal flow patterns on Doppler.

Serum bilirubin concentration gradually falls to normal levels during the first week. Aspartate aminotransferase (AST) and alanine aminotransferase (ALT) peak during the first three days and slowly level off, from then, in case of a healthy uptake of the grafted liver. Gamma glutamyl transferase and alkaline phosphatase, which are canalicular enzymes rise to four to five times of normal and then return to normal in the next few days. Synthetic functions normalize after the third day. While all of the above parameters may remain equivocal, the deteriorating clinical condition with multi-organ dysfunction may be the main clue to the nonfunction of transplanted liver. In such situations, liver biopsy (percutaneous or transjugular) may provide the ultimate answer.

Hyperacute graft rejection is very rare in liver transplantation and occurs due to the presence of preformed antibodies. On the other hand, acute cellular rejection is as common as 15-25%.[2,3] It can be present from within few days to a few years, and so in reality the term acute is inaccurate. Here a rise in serum bilirubin is associated with rise in aminotransferases and canalicular enzymes. Clinical symptomatology can be rather nonspecific with loss of appetite, pruritis, and fever without tachycardia. This picture is associated with increased hepatic artery resistive index on Doppler. The diagnosis is made on liver biopsy and treatment based on severity or the degree of rejection (Banff score).[4,5] The so called “chronic” rejection can also occur at any time and is evidenced by cholestatic features clinically and advancing arteriopathy and degenerating bile ducts on liver histology, with terminal liver failure ensuing eventually. Chronic rejection is extremely uncommon, accounting for less than 5%[6] of all cases of graft loss, and may occur due to untreated acute rejection, noncompliance to immunosuppression medication, or some immunological mechanisms which are not very well understood.

Various kinds of anastomotic problems can be present in the early postoperative days with varying incidence. Hepatic artery thrombosis, with incidence of 4-12%,[7] can present as sudden deterioration in hemodynamics, ARDS, severe coagulopathy, sudden and marked elevation of aminotransferases, with commonly accompanying liver abscesses due to bile duct strictures. Complications involving the portal vein are seen in 1.7-6% of liver transplant recipients.[8–10] Persistent ascites, enteric congestion and bleeding denoting portal hypertension, and later variceal hemorrhage may point to portal vein thrombosis. Doppler ultrasound followed by a traditional angiogram or magnetic resonance angiogram (MRA) will be diagnostic. Appropriate surgical or radiological intervention can be both, graft and life saving in these conditions. Patency of biliary tract can be jeopardized, either due to direct insult to the duct system or because of feeder vessel obstruction. Biliary tract complications account for up to 15% of postoperative surgical complications.[11] These are more common in partial grafts than in whole liver grafts. Again surgical intervention and/or radiological intervention are imperative.

Mediators from the liver or intestine may lead to a reperfusion syndrome after the graft is revascularized, which may manifest as hypotension from peripheral vasodilation, bradycardia, hyperkalemia, and pulmonary hypertension.

## Systemic Management

### Cardiovascular system

An important aspect of pretransplant workup is to establish suitability of a patient to withstand the severe cardiopulmonary stress that the surgery poses to him. With the upper age limit of recipients being increasingly liberalized, the possibility of coronary artery disease in the recipient must be kept in mind. Since cirrhotic patients have a modified lifestyle due to chronic and debilitating disease, as well as have a hyperdynamic systemic circulation, the classical symptoms of coronary insufficiency are often not present on presentation. This obviously does not imply an absence of the underlying cardiac disorder, which manifests in the intraoperative or early postoperative period, complicating the anesthetic and early ICU management.

Some patients, especially those with alcohol related cirrhosis, may present with reversible dilated cardiomyopathy after liver transplantation.[12] The intensivist must be aware of the possibility of perioperative myocardial infarction causing left ventricular dysfunction. Preoperative dobutamine stress echo (DSE) is a good screening test for occult coronary artery disease because it assesses the adequacy of myocardial oxygen supply. In addition, assessment must be made of valvular function and the presence of intrapulmonary shunting (by contrast Echo) and portopulmonary hypertension. A negative DSE predicts a good prognosis, that is, a low probability of perioperative cardiac events.[13] Unrecognized coronary artery disease is associated with a mortality of up to 50% and morbidity of 80%.[14] While high cardiac output is common in cirrhosis, a relative low cardiac output status may be seen in cirrhotic patients with cardiomyopathy and amyloidosis. These patients will need advanced hemodynamic monitoring such as Swan Ganz or PiCCO, and inotropic support.

Arterial hypotension is quite common in peritransplant period. A vasodilated and hyperdynamic state is typical of liver failure. These changes resolve slowly after liver transplantation. Magnitude of hepatic reperfusion syndrome may influence the posttransplant cardiopulmonary status. It may take days to weeks sometimes for these changes to revert to near normal. Failure to normalize with reduction in the level of vasoconstrictor support indicates poor prognosis. Sepsis may further complicate the picture. Elevated venous pressures will lead to hepatic congestion, and this may in turn increase the portal pressure. As a result of this, graft function may further suffer and lead to bacterial translocation and endotoxemia. To avoid such sequences of events, hypotension has to be classified as cardiac or vasodilatory. Moderate filling followed by vasoconstriction should treat this. Whereas, inotropic support can be provided by dobutamine and adrenaline, vasoconstriction can be achieved with low dose noradrenalin or vasopressin. Cardiac tamponade has to be excluded in face of low cardiac output and high filling pressures.

Systemic hypertension is common in the early postoperative period in a patient with a well functioning graft. It generally occurs either due to lack of analgesia or sedation in this setting. Later in the course of patient recovery, hypertension occurs due to cyclosporine or tacrolimus. Treatment is usually initiated when the systolic blood pressure is greater than 160 mmHg or the diastolic blood pressure greater than 100 mmHg. Atrial fibrillation may occur due to perioperative fluid shifts, acid base imbalance and electrolyte abnormalities. Treatment includes etiological management, beta-blockers and calcium antagonists, but amiodarone should be avoided if possible because of its potential hepatotoxicity.

### Pulmonary system

It is estimated that 45-69% patients with cirrhosis have some degree of hypoxemia.[15] Long-standing mechanical factors like ascites, atelectasis, and pleural effusion with restrictive lung changes are added to the major post upper abdominal surgical status leading to hypoxemia in the posttransplant period. Good pain control, chest physiotherapy, and incentive spirometry with functioning graft will improve this scenario.

Widespread vasodilatation with vascular shunting in liver disease will have its reflection on the pulmonary system. In a more etiopathogenic sense, hepatopulmonary syndrome (HPS) and porto-pulmonary hypertension (PPH) may continue into the posttransplant period. Introduction of pulmonary artery catheter is essential for management of these patients. HPS is hypoxemia in the background of liver disease with air contrast echocardiogram (Bubble study) demonstrable intrapulmonary shunting. This syndrome presents with dyspnea and desaturation on erect posture, platypnea and orthodeoxia. Their ventilation-perfusion mismatch resolves after a few days of successful transplantation.[16] Fixed nonreversible shunt denotes poor prognosis. Pulmonary hypertension is more likely in cirrhotic patients with worsening porto-pulmonary shunting. This can affect right ventricular function and may have to be corrected with epoprostenol (prostacylclin-PG1_2_), which is a potent pulmonary and systemic vasodilator. Severe PPH is a contraindication to transplantation. Pulmonary hypertension developing first time after transplant is usually due to pulmonary embolism.

Cardiogenic and noncardiogenic pulmonary edema, Acute Respiratory Distress Syndrome (ARDS), and pulmonary infection are not uncommon in this period. In the immediate postoperative period, OLT recipients may develop ARDS as a result of the surgical insult or transfusion related acute lung injury[17] (TRALI). In case of a suspected infection, broncho-alveolar lavage has to be obtained for quantitative bacteriological and fungal cultures, which should be followed by sensitive antibiotics or antifungal agents. Early application of noninvasive ventilation rather than just supplemental oxygen can reduce the incidence of reintubations, major or fatal complications and overall mortality. Preventing intubation should be a major aim of management of respiratory failure in these immunocompromised patients.

Ventilatory strategies that minimize insult to the graft function should be used, as positive-pressure ventilation and high positive end-expiratory pressures may alter the splanchnic blood flow and decrease graft oxygenation and cause congestion of the inferior vena cava and hepatic vein drainage areas. Posttransplant ventilation is usually for a day or two, depending on various pulmonary and extra-pulmonary determinants. This is a risk-benefit ratio evaluation between the need for good graft oxygenation and risk of infection. All possible precautions should be employed to avoid ventilator-associated pneumonia.

### Renal system

Pretransplant renal dysfunction is an independent predictor of posttransplant morbidity and mortality.[1] Up to 25% of recipients suffer from renal impairment prior to transplantation, and nearly two-thirds of transplant recipients show impaired posttransplant renal function.[18] It has been found that in the posttransplant period there is a 40% decline in the glomerular filtration rate at the end of 6 weeks after which it stabilizes.[19]

The etiological reasons for renal dysfunction in the posttransplant phase include pre-transplant renal dysfunction, which maybe due to acute tubular necrosis (ATN) or hepato-renal syndrome or other medical problems, tubular damage due to peritransplant hypotension, graft dysfunction, ATN from postoperative sepsis and drug induced injury (cyclosporine, tacrolimus, amphotericin, aminoglycosides etc.). Management of renal dysfunction depends on the etiology. Those with HRS are more likely to require renal replacement therapy (RRT) with about 10% progressing to develop ESRD.[20] Intra-operative management of hypotension, use of veno-venous bypass,[21] and avoidance of nephrotoxic drugs are important reno-protective strategies. Nephrotoxicity is a known complication of calcineurin inhibitors (CNI) used to prevent rejection. Reducing the dosage, use of CNI sparing anti-rejection protocol or delaying introduction of CNIs in those with high probability of renal dysfunction and use of calcium channel blockers in CNI related hypertension have been found to be useful strategies in long term renal protection. Oliguria may be the earliest warning sign of renal dysfunction. 8-10% of transplant recipients require renal replacement therapy in the immediate post op period.[22,23] Dialysis, preferably lactate free continuous renal replacement therapy (CRRT) is required to stabilize these patients. So-called reno-protective agents like dopamine, calcium channel blockers or prostaglandins have not been proven to be of value. Combined liver and kidney transplant is an option reserved for those patients with pre-transplant renal dysfunction due to other concomitant medical illness or intrinsic renal disease.

### Gastrointestinal system

Many patients are severely malnourished before transplantation. As most patients have brisk return of gastrointestinal function, early enteral nutrition is the goal, except in patients with choledochojejunostomy. Upper gastrointestinal bleed is usually due to gastritis or stress ulceration. In general, upper gastrointestinal bleeding and good graft function do not co-exist. Portal vein thrombosis may result in recurrence of varices and bleeding. Posttransplant pancreatitis is a feared complication of liver transplantation. Conservative management is usually preferred to aggressive measures.

### Central nervous system

A functioning allograft will generally improve neurological impairment, especially in those patients with certain pre-existing metabolic encephalopathy. Neurological events do occur, which can range from seizures to stroke to coma and are often first recognized while the patient is still in the intensive care unit. Clinical series have documented neurological complications in 8.3% to 47% of all patients receiving liver transplantation.[24,25]

Alteration of mental status is common and upto one-third of liver recipients can have some degree of neurologic dysfunction in the perioperative period.[26] Rapid recovery from encephalopathy is expected in the presence of good graft. Compromised graft function may result in recurrence of encephalopathy. Fulminant hepatic failure patients undergoing liver transplant need continuous monitoring of intra-cranial pressure. Focal deficit should lead to suspicion of stroke or embolism. Acute change in mental status and occurrence of seizures should need checking up of drug, electrolytes and blood glucose levels.

Psychosis is another feared complication in transplant recipients. It has a multifactorial etiology and could be due to prolonged ICU stay, use of steroids and other immunosuppressants. The fact that most antipsychotics are hepatotoxic is a major impediment in the treatment of this condition. Psychosis resulting in a noncompliant patient, can be a major stumbling block in rapid recovery, due to ineffective delivery of medication, physiotherapy and mobilization.

### Endocrine and metabolic problems

Hyperglycemia is common because of surgical stress, steroid administration and insulin resistance associated with liver failure and there is increasing evidence to deploy tight control regimens.[27] Hypoglycemia may be a sign of inadequate graft function or severe sepsis.

Hypothermia is common in the posttransplant patient and can precipitate metabolic acidosis, and accentuate coagulopathy. Mild metabolic acidosis is common in the first few hours after transplantation. Optimum fluid and ionotropic management should attenuate this acidosis. Persistent metabolic acidosis in the absence of other causes should warrant suspicion of graft dysfunction. Slightly delayed acidosis may indicate sepsis. Serial lactate levels are helpful in managing such situations.

Adrenal insufficiency and hypothyroidism are sometimes seen in these patients and have to be corrected after establishing the diagnosis.

### Fluid and electrolytes

The recipient is generally kept in a euvolemic or slightly hypovolemic state in the posttransplant period, with minimal intravenous infusions, to optimize graft function and avoid pulmonary edema. If needed, 5% dextrose with 0.45% NS is used unless the serum sodium is less than 130 mEq/L, when 5% dextrose with 0.9% NS can be used. Gelatins are generally preferred to starches in these patients. Packed red cell and albumin transfusions are preferred when volume expansion is required.

Electrolyte imbalance is quite common in these patients. Hyponatremia should be gradually corrected with judicious use of fluids with a target serum sodium rise of <10-12 mEq/dL/day is desirable.[28] Hypomagnesemia is common in cirrhotic patients and may be exacerbated in the posttransplant patient by excessive blood loss and medications (CNIs, loop diuretics, and amphotericin B). Recovering graft has a high requirement for phosphate and magnesium and these should be replaced adequately. Ionized serum calcium levels should be monitored as total calcium levels depend on the albumin concentration which may fluctuate widely in the early posttransplant period. Pretransplant hypocalcemia due to malnutrition and vitamin D dysfunction may be exacerbated early in the posttransplant patient by citrate chelation (with blood transfusion), gastrointestinal malabsorption and hepatocyte injury resulting in an intracellular shift of calcium. Hypercalcemia and hypermagnesemia are rare.

### Coagulopathy

Coagulopathy results from preexisting portal hypertension, inadequate clotting factor synthesis, hypersplenism, fibrinolysis, hypocalcaemia and dilution. The risk of bleeding must be balanced against the risk of hepatic artery or portal vein thrombosis, so over correction should be avoided. Hence, monitoring of coagulation becomes mandatory after liver transplantation. Thrombelastography (TEG), a method for evaluating the viscoelastic properties of the blood clot, can be used to complement the standard coagulation parameters in these patients. TEG can be useful in differentiating between bleeding secondary to incomplete surgical hemostasis, platelet dysfunction or anomalies in coagulation factors and therefore, can help in optimizing and minimizing blood component usage by guiding use of selective blood component therapy.[29] TEG guided replacement can reduce transfusions and attendant complications.[30,31] It may also be useful in detecting a hypercoaguable state, not reflected by standard coagulation parameters, which may be present after any major surgery and thus, may guide antithrombotic therapy with increased safety.[32,33] TEG has further advantages of allowing rapid bed-side monitoring and may be useful in assessing the graft function.[34]

Platelet dysfunction due to renal insufficiency can be managed with desmopressin. Replacement of blood products is necessitated in the presence of active bleeding or any planned intervention. Otherwise, maintenance of an INR between 1.5 and 2, a platelet count >50 × 10^9^/L and a fibrinogen level >100 mg/dL is satisfactory.

### Infection

The primary cause of death after liver transplantation is infection.[35] Bacterial and fungal infections are common in the early posttransplant period, originating from intravascular lines, lung, urinary tract, surgical wound, and the biliary system.[36] Prophylaxis against gram-negative bacteria is usually deployed depending on local antibiogram patterns. Prolonged surgery, multiple transfusions, malnutrition, hyperglycemia, requirement of dialysis and retransplantation are risk factors for fungal infections.[37] Viral infections are seen much later in this population. A detailed account of posttransplant infections is beyond the preview of this article [Table 1].

**Table 1:** Common infections in the posttransplant period[36,38–40]

Early postoperative period (<30 days)	Late postoperative period (>30 days)
Bacteria	
Nosocomial infections (coagulase-negative, *Staphylococcus aureus, gram negative organisms-enterobacter, pseudomonas)*	Salmonella *Streptococcus pneumoniae Hemophilus influenzae*
* Clostridium difficile*	Nocardia
Legionella	Listeria
Fungus	
*Candida*	Aspergillus
*Aspergillus*	Cryptococcus
	Reactivation of endemic mycoses (histoplasmosis and coccidiomycosis)
Viral	
Reactivation of hepatitis B and C	Cytomegalovirus
Reactivation of Herpes simplex virus	Epstein-Barr virus
	Reactivation of hepatitis B and C
	Adeno virus
	Varicella zoster virus
Others	
	Tuberculosis
	*Pneumocystis carinii* pneumonitis

## Immunosuppressive Therapy

Triple therapy is generally given in most centers based on a CNI, like tacrolimus or cyclosporin, in conjunction with an antiproliferative agent (mycophenolate mofetil) and a steroid.[41] A dual regimen of steroids and a CNI has shown to be equally efficacious as triple therapy.[42] The advantage of early use of triple therapy is that it may allow delaying the initiation of the CNI, while the posttransplant changes in renal function recover. One must maintain a balance between under-immunosuppression, which may lead to graft rejection, and over-immunosuppression, which may lead to sepsis and malignancy.

Cyclosporin and tacrolimus seem to be similar in terms of graft and patients survival.[43] However, tacrolimus is associated with fewer episodes of rejection and less need for steroid use. Tacrolimus is equally nephrotoxic and is associated with increased rates of diabetes and neurotoxicity but has a lower incidence of hypertension and hyperlipidemia.[44,45]

## Nutritional Support

Factors such as preoperative malnutrition, stress from surgery, and immunosuppressive therapy, enhance the need for nutritional support after transplantation. In the immediate postoperative period, protein catabolism is markedly increased,[46] and hence, these patients should receive 1.5-2.0 grams of protein per kilogram of dry weight during this phase.[47] Energy requirements are not significantly elevated; especially in an uncomplicated, nonseptic patient therefore, calories should be provided at approximately 120-130% of the calculated basal energy expenditure (BEE).[47] Patients should be encouraged to start oral diets as soon as tolerated.

## Conclusion

The principles guiding critical care for liver transplant patients, are to provide favorable circumstances for the new liver to start functioning and maintain the function of other organs to aid in this process.
